# PKD2/polycystin-2 inhibits LPS-induced acute lung injury in vitro and in vivo by activating autophagy

**DOI:** 10.1186/s12890-023-02449-w

**Published:** 2023-05-18

**Authors:** Fan Pan, Lina Bu, Kaixuan Wu, Aizhong Wang, Xiaotao Xu

**Affiliations:** Department of Anesthesiology, Affiliated Shanghai Sixth People’s Hospital, Shanghai Jiao Tong University, Shanghai, 200233 China

**Keywords:** Polycystin-2, Acute lung injury, Inflammation, Autophagy, LPS

## Abstract

Polycystin-2 (PC2), which is a transmembrane protein encoded by the PKD2 gene, plays an important role in kidney disease, but its role in lipopolysaccharide (LPS)-induced acute lung injury (ALI) is unclear. We overexpressed PKD2 in lung epithelial cells in vitro and in vivo and examined the role of PKD2 in the inflammatory response induced by LPS in vitro and in vivo. Overexpression of PKD2 significantly decreased production of the inflammatory factors TNF-α, IL-1β, and IL-6 in LPS-treated lung epithelial cells. Moreover, pretreatment with 3-methyladenine (3-MA), an autophagy inhibitor, reversed the inhibitory effect of PKD2 overexpression on the secretion of inflammatory factors in LPS-treated lung epithelial cells. We further demonstrated that overexpression of PKD2 could inhibit LPS-induced downregulation of the LC3BII protein levels and upregulation of SQSTM1/P62 protein levels in lung epithelial cells. Moreover, we found that LPS-induced changes in the lung wet/dry (W/D) weight ratio and levels of the inflammatory cytokines TNF-α, IL-6 and IL-1β in the lung tissue were significantly decreased in mice whose alveolar epithelial cells overexpressed PKD2. However, the protective effects of PKD2 overexpression against LPS-induced ALI were reversed by 3-MA pretreatment. Our study suggests that overexpression of PKD2 in the epithelium may alleviate LPS-induced ALI by activating autophagy.

**Supplementary Information:**

The online version contains supplementary material available at 10.1186/s12890-023-02449-w.

## Introduction

Acute lung injury (ALI) and acute respiratory distress syndrome (ARDS) are characterized by a serious inflammatory response and refractory hypoxemia resulting from alveolar-capillary barrier damage and noncardiogenic pulmonary oedema [1, 2]. Disruption of the alveolar-capillary membrane, neutrophil migration, and the release of a massive amount of proinflammatory cytokines are cellular characteristics of ALI [3]. The epithelium is the site of the primary host defence in response to non-infectious and infectious stimulation during both the pathogenesis and resolution of ALI [4, 5]. Lipopolysaccharide (LPS) has been widely used to establish a clinically relevant model of ALI. Decreasing the bacterial load and alleviating organ damage caused by excessive inflammation are the main therapeutic strategies for ALI. However, despite mechanical ventilation protocols and improvements in critical care, the mortality of ALI remains high [4]. Therefore, novel approaches for ALI treatment and prevention are needed.

PKD2/PC2/TRPP2 (polycystin 2, a transient receptor potential cation channel) belongs to the transient receptor potential (TRP) superfamily of ion channels, which form nonselective cation channels on the cell membrane and permit the passage of divalent cations to various degrees [6, 7]. TRP channels are involved in a variety of cellular functions, from osmosis and mechanical transduction to the regulation of Ca2^+^ homeostasis and vascular regulation. In terms of subcellular localization, PKD2 is expressed at the cell membrane and in the endoplasmic reticulum (ER), protocilia, centrosomes, and spindles of dividing cells [8]. PKD2 was found to be present in the primary cilia of human type II alveolar epithelial A549 cells, calu-3 cells and rat bronchioles [9]. Numerous studies have demonstrated that PKD2 plays a key role in cell fate, especially in cell differentiation, proliferation, survival, apoptosis, and autophagy [10]. Autophagy, an evolutionarily conserved cellular process that affects many aspects of innate and adaptive immunity in almost all human cell types, controls the inflammatory response in some diseases [11, 12]. Some recent evidence has indicated that autophagy activation can attenuate LPS-induced ALI [13].

To explore whether overexpression of PKD2 protects against LPS-induced ALI by activating autophagy, we established A549 cells expressing Ad*PKD2* and evaluated the molecular mechanism of PKD2. Finally, we found that PKD2 prohibited LPS-induced ALI in mice infected with a recombinant adenovirus expressing *PKD2*.

## Materials and methods

### Cell culture and in vitro transfection

Pulmonary epithelial A549 cells (ATCC, USA) were incubated in F-12 medium (Gibco, Life Technology, USA) supplemented with 10% foetal bovine serum (FBS, Gibco) in a humidified incubator at 37 °C with 5% CO2. A recombinant adenovirus containing the mouse *PKD2* gene was purchased from GeneChem Company (Shanghai GeneChem Co., Ltd., Shanghai, China). An adenovirus without transgene expression was used as a negative control (AdCo). According to the manufacturer’s instructions, the optimal transfection titre was obtained in a preliminary experiment with A549 cells transfected with adenovirus, and this value was used for subsequent transfection experiments. After successful transfection, A549 cells were stimulated with LPS (50 µg/ml) [14].

### In vivo PKD2 transfection and animal models

Animals were housed in an environment controlled for temperature (23 °C), humidity (50%), and light (12-h dark-light cycles). The mice were allowed free access to aseptic food and water and were acclimated for 1 week before the experiments. Three days before the animal model was established, the mice were anaesthetized using 2% sodium pentobarbital (80 mg/kg, Sigma-Aldrich, St. Louis, MO, USA) by i.p. injection and given control adenovirus (AdCo) or Ad*PKD2* (5 × 10^12^ vg/ml) in 30 µl of PBS via intratracheal (i.t.) administration to induce the overexpression of PKD2 in the lung epithelium. Twelve mice were randomly divided into four groups (n = 3 per group): the AdCo group, AdCo + LPS group (AdCo + LPS), Ad*PKD2* + LPS group (Ad*PKD2* + LPS), and Ad*PKD2* + 3-methyladenine (3-MA) (15 mg/kg) + LPS group. For the control group, normal saline was administered to the mice by i.t. injection. For the AdCo + LPS group and AdPKD2 + LPS group, 1 mg/kg LPS was administered to the mice by i.t. injection three days after i.t. injection of the adenovirus. For the Ad*PKD2* + 3-MA (15 mg/kg) + LPS group, 1 mg/kg LPS was administered by i.t. injection three days after i.t. injection of the adenovirus, and 3-MA was administered intraperitoneally 30 min before LPS treatment. The doses of LPS and 3-MA used in this study were based on a previous study [15, 16]. Twelve hours after LPS treatment, the mice were sacrificed by an i.p. injection of 50 mg/kg pentobarbital. From each mouse, the left lung was harvested for HE staining, and the rest of the lung tissues were taken for ELISA and western blotting.

### Pulmonary histopathology analysis

The lung tissues were fixed in 4% formaldehyde, embedded in paraffin, and cut into 5-µm-thick sections. Haematoxylin and eosin staining of the slides was performed, followed by visualization. Five microscopic fields were used to evaluate the lung injury score based on a previous protocol [17].

### Western blot analysis

Western blot analysis was performed as previously described [18]. Equal amounts of protein (15–30 µg) were loaded onto 12–15% SDS-polyacrylamide gels and transferred to PVDF membranes. The membranes were blocked with 5% skim milk for 1 h at room temperature and incubated with anti-PKD2 (Santa Cruz, sc-1745), anti-β-actin (CST,D6A8), anti-GAPDH (CST,14C10), anti-Beclin-1 (CST,3738 S), anti-LC3B (HuaBio,JJ090-6), and anti-SQSTM1/P62 (abcam, ab13524) antibodies overnight at 4 °C. After washing with TBST, the membranes were incubated with HRP-conjugated secondary antibody at room temperature for 1 h. Proteins were detected using an ImageQuant LAS 4000 mini with an enhanced chemiluminescence (ECL) detection kit (Millipore, USA), and band densities were quantified using ImageJ software (version 4.0.0, USA).

### RNA extraction and qRT‒PCR

Total RNA was extracted from A549 cells using TRIzol reagent (BioTNT, China) following the manufacturer’s instructions. One microgram of RNA was used for reverse transcription to synthesize cDNA, which was used as a quantitative real-time PCR template. The mRNA levels of TNF-α, IL-1β and IL-6 were measured with a SYBR Green Master Mix kit (Roche) according to the manufacturer’s instructions and standardized against β-actin, which was used as a loading control. Real-time PCR.

amplification was performed as follows: initial denaturation at 95 °C for 30s; 40 cycles of denaturation at 95 °C for 3 s, annealing at 60 °C for 30 s, and extension at 72 °C for 10 s; one cycle of 95 °C for 15 s; 65 °C for 1 min; and a final cooling step at 40 °C for 30 s. The 2^−ΔΔCt^ method was used to calculate the expression level of mRNA. The following primer sequences were used for amplification: TNF-α, forward:TGACTTGTTCCTCAGCCTCTT, reverse:TGAGGTACAGGCCCTCTGAT; IL-1β, forward:CGATGCACCTGTACGATCAC, reverse:TCTTTCAACACGCAG GACAG;IL-6, forward:TGCGTCCGTAGTTTCCTTCT, reverse:GGAATCTTCTCC TGGGGGTA; β-actin, forward:TGGCACCCAGCACAATGAAGATCA, reverse:

CTGCTTGCTGATCCACATCTGCT.

### ELISA

The levels of the cytokines IL-6, IL-1β, and TNF-α in the lung tissue were quantitatively measured by enzyme-linked immunosorbent assay (ELISA) (R&D Systems, Minneapolis, MN, USA) as described previously [19].

### CCK-8 cell viability assay

A549 cells were treated as described in the experimental scheme, seeded at a density of 3 × 10^4^ cells/well in 96-well plates and kept overnight for 24 h at 37 °C under 5% CO2. Three duplicate wells were used for each experimental group. Afterwards, 10 µl of CCK-8 reagent was added to each well, and the mixtures were continuously incubated at 37 °C for 2 h. The absorbance values at 450 nm were detected using an automatic porous spectrophotometer (Molecular Devices, USA).

### Determination of the lung W/D weight ratio

The lung wet/dry (W/D) ratio was used to evaluate lung oedema. The fresh upper portion of the left lung was excised, rinsed briefly in PBS, blotted and weighed to determine the wet weight. Then, each of the lungs was dried for 72 h at 80 °C in an oven and weighed to measure the dry weight [20].

### Myeloperoxidase (MPO) assay

Lung tissues were homogenized, and the supernatant was collected and subjected to an MPO activity assay using a commercially available kit (Nanjing Jiancheng Bioengineering Institute, Nanjing, China). MPO activity was determined by measuring the absorbance at 460 nm [21].

### Statistical analyses

All statistical analyses were performed using GraphPad Prism 8.0 (GraphPad Software, San Diego, CA). All values represent the mean ± SEM. Two-tailed Student’s t test or one-way ANOVA followed by Tukey’s test was used for statistical analyses. Differences were estimated to be significant at *P* < 0.05.

## Results

### Overexpression of PKD2 protected the viability of lung epithelial cells under LPS treatment

**Fig. 1:**
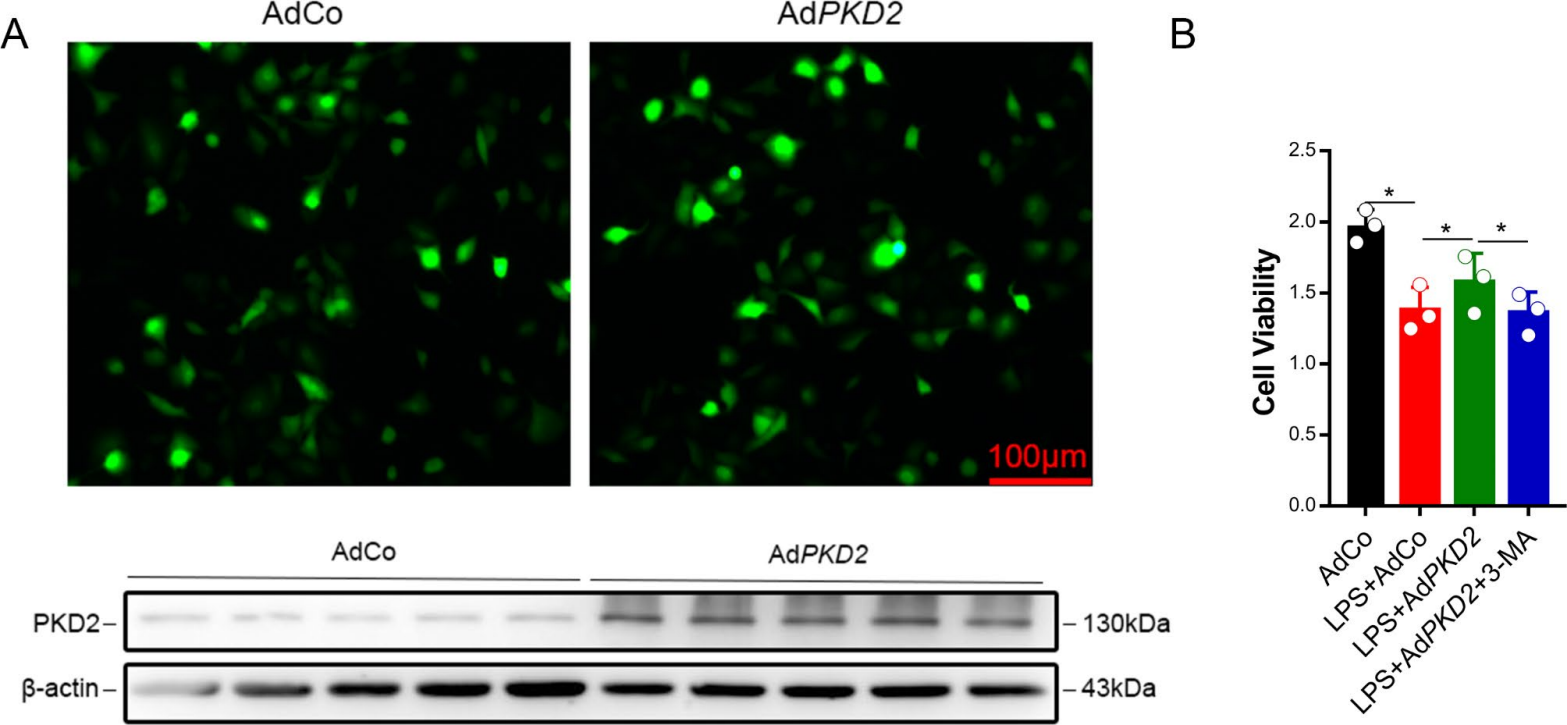
Overexpression of PKD2 protected the viability of lung epithelial cells treated with LPS. (A) A549 cells were transfected with AdCo or Ad*PKD2* for 48 h. The transfection efficiency was detected by fluorescence microscopy (scale bar: 100 μm), and the expression of PKD2 was determined by western blotting. (B) A549 cells were transfected with AdCo or Ad*PKD2* for 48 h. Then, the cells were stimulated with LPS (50 µg/ml) for 12 h or pretreated with the autophagy inhibitor 3-MA (1 mol/L) for 15 min, followed by LPS (50 µg/ml) treatment for 12 h. The viability of the A549 cells was detected by CCK-8 assay. The experiments were repeated three times (three independent experiments). The data shown in the graphs are the mean ± SEM. *P < 0.05

To explore the effect of PKD2 on cell viability, lung epithelial cells (A549 cells) were treated with LPS. A549 cells were transfected with AdCo or Ad*PKD2* for 48 h. The transfection efficiency was detected with a fluorescence microscope. The western blot results demonstrated that PKD2 levels in the A549 cells were markedly increased in the Ad*PKD2* group compared to the AdCo group (Fig. 1A). Forty-eight hours after transfection, the cells were incubated with 50 µg/ml LPS for 12 h, and we found that the viability of the A549 cells was significantly decreased in the AdCo group, but the overexpression of PKD2 could increase the viability of the A549 cells. To further explore whether autophagy was involved in the ability of PKD2 overexpression to increase the viability of the A549 cells, an autophagy inhibitor, 3-MA (1 mol/L), was added 15 min before LPS administration. The results showed that 3-MA treatment reversed the effect of PKD2 overexpression on cell viability (Fig. 1B). The above results demonstrated that overexpression of PKD2 had an inhibitory effect on LPS-induced lung epithelial cell injury.

### PKD2 inhibited the LPS-induced inflammatory response in lung epithelial cells

**Fig. 2:**
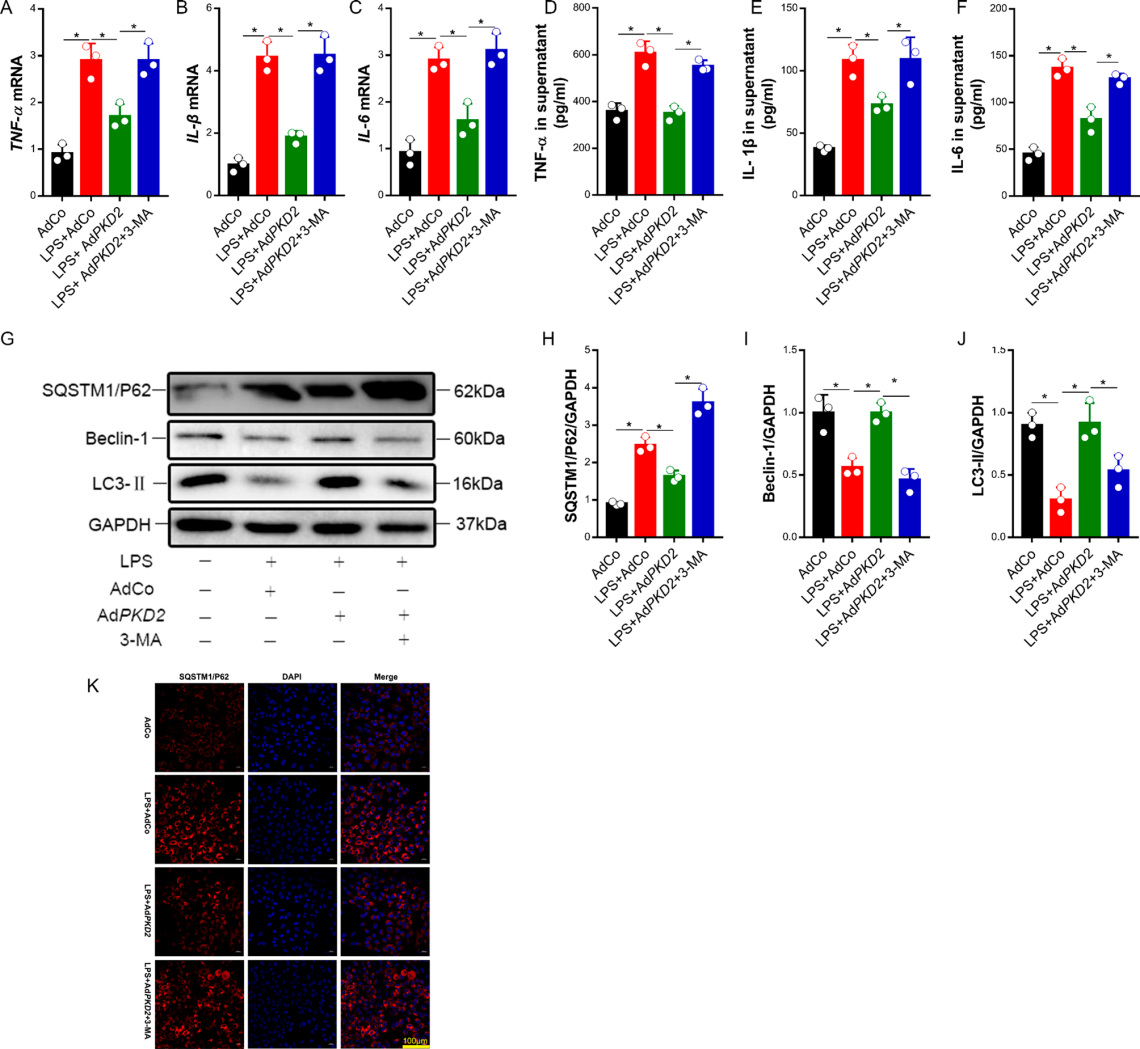
PKD2 decreased the inflammatory response by activating autophagy. A549 cells were transfected with AdCo or Ad*PKD2* for 48 h. Then, the cells were stimulated with LPS (50 µg/ml) for 12 h or pretreated with the autophagy inhibitor 3-MA (1 mol/L) for 15 min, followed by LPS (50 µg/ml) treatment for 12 h. (A-C) The TNF-α, IL-1β and IL-6 mRNA levels in the cells were measured by qRT‒PCR. (D-F) The expression of TNF-α, IL-1β and IL-6 in the cells was detected by ELISA. (G-I) The levels of SQSTM1/P62 and Beclin-1 and the LC3-II were detected by western blot analysis. (J) Representative immunofluorescence images of SQSTM1/P62 (red) and DAPI (blue) double staining in A549 cells. Scale bar = 100 μm. (K) Representative immunofluorescence images of SQSTM1/P62 (red) and DAPI (blue) double staining in A549 cells. Scale bar = 100 μm. The experiments were repeated three times (three independent experiments). The data shown in the graphs are the mean ± SEM. *P < 0.05

To investigate the effect of PKD2 on inflammatory cytokine production in LPS-treated lung epithelial cells, A549 cells were transfected with AdCo or Ad*PKD2*. Forty-eight hours after transfection, the cells were incubated with 50 µg/ml LPS and/or 1 mM 3-MA. The overexpression of PKD2 decreased the levels of TNF-α, IL-1β, and IL-6, but the inhibitory effects of PKD2 overexpression were reversed by 3-MA in the LPS-treated A549 cells (Fig. 2A-F). These data indicated that overexpression of PKD2 restrained LPS-induced inflammatory responses in vitro.

### Overexpression of PKD2 decreased LPS-induced lung epithelial cell injury by activating autophagy

To explore whether the protective effect of PKD2 overexpression against LPS-induced lung epithelial cell injury was associated with autophagy activation, the levels of several key autophagy-related proteins were detected using western blotting. LPS treatment significantly decreased the LC3-II and Beclin-1 level and increased the level of SQSTM1/P62. Overexpression of PKD2 increased the level of LC3-II and decreased the level of SQSTM1/P62 in LPS-treated A549 cells. The effect of PKD2 overexpression on LPS-induced changes in autophagy-related protein expression was reversed by 3-MA (Fig. 2G-J). In addition, immunofluorescence analysis showed decreased SQSTM1/P62 levels in the PKD2 overexpression + LPS group compared with the LPS group. The effect of PKD2 overexpression on the LPS-induced increase in SQSTM1/P62 was counteracted by 3-MA (Fig. 2K). The above results indicated that overexpression of PKD2 mitigated LPS-induced lung epithelial cell injury by activating autophagy in vitro.

### PKD2 blunted LPS-induced lung inflammatory injury through autophagy activation

**Fig. 3:**
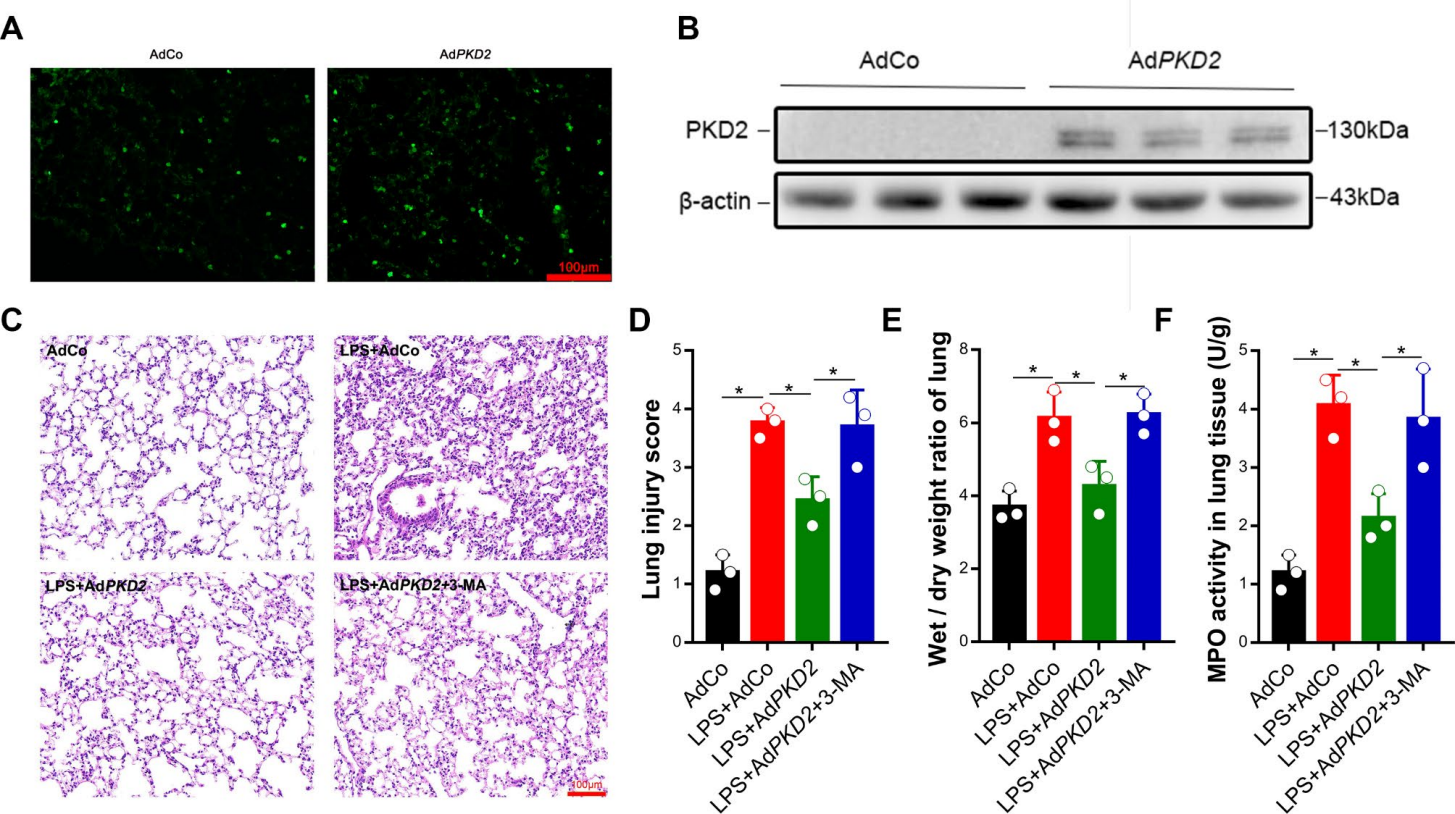
PKD2 alleviated LPS-induced inflammatory injury in the lung. Mice were intratracheally injected with adenovirus expressing PKD2 or GFP (5 × 10^12^ vg/ml) in 30 µl of PBS for three days. (A and B) The transfection efficiency was detected by fluorescence microscopy (scale bar: 100 μm), and the expression of PKD2 was determined by western blotting. Mice were intratracheally injected with adenovirus expressing *PKD2* or *GFP* (5 × 10^12^ vg/ml) in 30 µl of PBS for three days. Then, the mice were intratracheally injected with LPS (1 mg/kg) for 24 h or intraperitoneally injected with the autophagy inhibitor 3-MA (15 mg/kg) for 30 min, followed by LPS (1 mg/kg) treatment for 24 h. (C) Histological evaluation of the lung samples was carried out using HE staining. (D) Lung injury scores were determined on the basis of four independent parameters. (E) The lung W/D ratio was detected. (F) MPO activity in the lung tissues was detected with an MPO activity kit. The experiments were repeated three times (three independent experiments). The data shown in the graphs are the mean ± SEM. *P < 0.05

We next explored the effect of PKD2 on LPS-induced lung injury. We generated mice overexpressing PKD2 by i.t. injection of adenovirus expressing *PKD2* or *GFP* into the lung tissue. The transfection efficiency was detected with a fluorescence microscope (Fig. 3A). The western blot results demonstrated that PKD2 levels in the lung tissue were markedly increased in the Ad*PKD2* group compared with the AdCo group (Fig. 3B). LPS (1 mg/kg) was administered by i.t. injection three days after i.t. injection of adenovirus, and 3-MA was administered 30 min before LPS treatment. Twelve hours after LPS treatment, LPS had led to severe inflammatory and histological changes in the lung tissues, including inflammatory cell infiltration, damage to the alveolar wall and pulmonary congestion, but these changes were markedly decreased in the group with *PKD2* overexpression. The effect of PKD2 on LPS-induced lung tissue histopathological injury was reversed by 3-MA (Fig. 3C and D). We also found that the lung W/D ratio and MPO activity, which reveal the level of inflammation, were increased in the LPS group. Importantly, the lung weight/dry ratio and MPO activity were markedly reduced in the group with *PKD2* overexpression. The effects of PKD2 on the lung weight/dry ratio and MPO activity were reversed by 3-MA treatment (Fig. 3E and F).

**Fig. 4:**
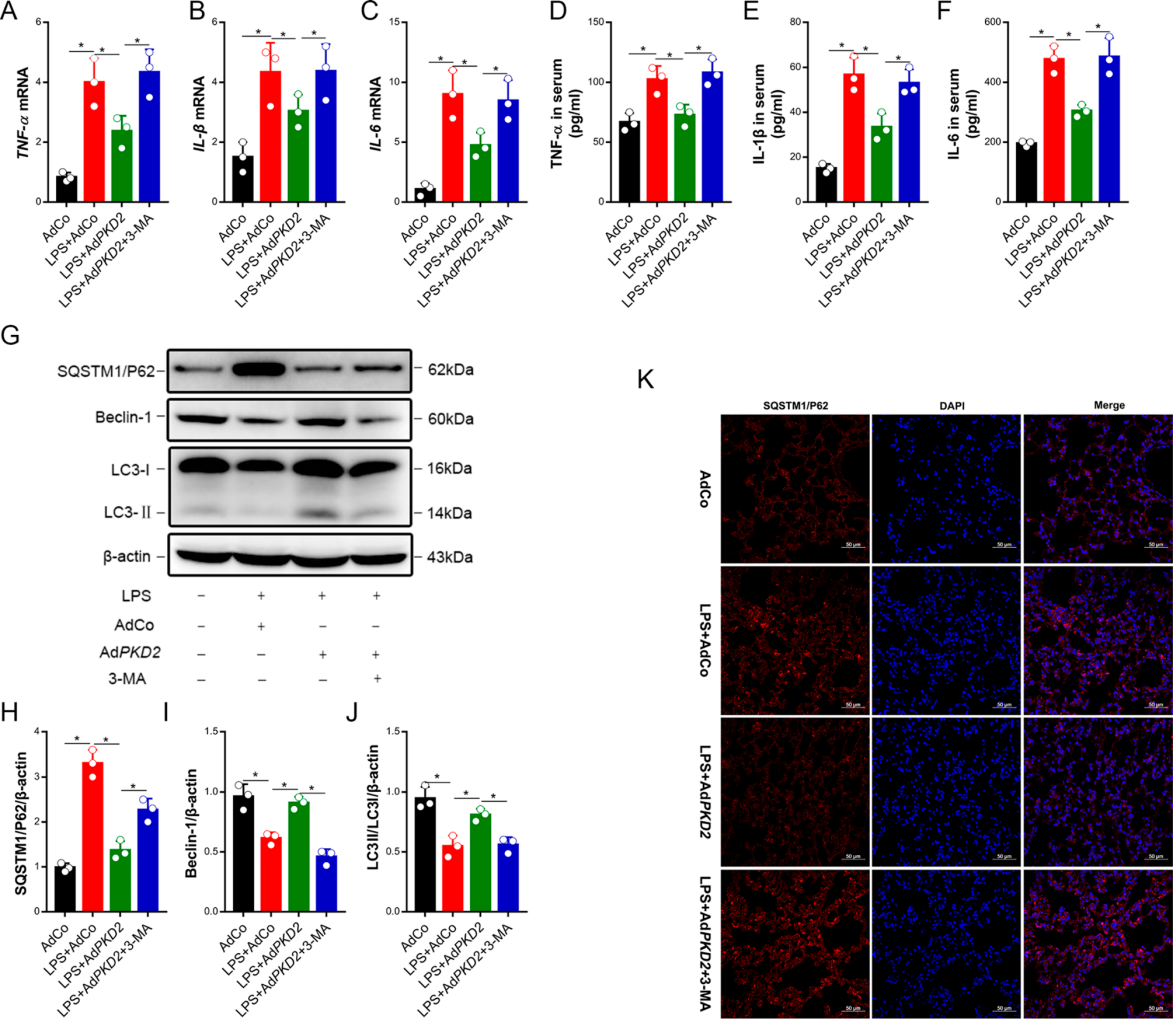
PKD2 mitigated LPS-induced lung injury by activating autophagy. Mice were intratracheally injected with adenovirus expressing *PKD2* or *GFP* (5 × 10^12^ vg/ml) in 30 µl of PBS for three days. Then, the mice were intratracheally injected with LPS (1 mg/kg) for 24 h or intraperitoneally injected with the autophagy inhibitor 3-MA (15 mg/kg) for 30 min, followed by LPS (1 mg/kg) treatment for 24 h. (A-C) The TNF-α, IL-1β and IL-6 mRNA levels in the lung tissues were measured by qRT‒PCR. (D-F) The expression of TNF-α, IL-1β and IL-6 in the lung tissues was detected by ELISA. (G-J) The levels of SQSTM1/P62 and Beclin-1 and the LC3-II/I ratio were detected by western blot analysis. (K) Representative immunofluorescence images of SQSTM1/P62 (red) and DAPI (blue) double staining in the lung tissue are shown. Scale bar = 100 μm. The experiments were repeated three times (three independent experiments). The data shown in the graphs are the mean ± SEM. *P < 0.05

To further evaluate the effect of PKD2 on the inflammatory response to ALI induced by LPS, we first detected the gene expression and secretion of TNF-α, IL-1β and IL-6 in the lung tissue, which were increased by LPS. However, the gene expression and secretion of TNF-α, IL-1β and IL-6 in the lung tissues with ALI were reduced by PKD2 overexpression, and the inhibitory effects of PKD2 against inflammatory factors in the lung tissues with ALI were attenuated by 3-MA (Fig. 4A-F). These results demonstrated that overexpression of PKD2 decreased lung inflammation in ALI by activating autophagy in vivo.

### The effect of PKD2 on autophagy-related protein levels in lung tissue with LPS-induced ALI

To elucidate the effect of PKD2 on autophagy-related proteins in lung tissue with LPS-induced ALI, we evaluated the protein ratio of LC3-II/I and the protein levels of Beclin-1 and SQSTM1/P62 by western blotting. As shown in Fig. 4G-J, the ratio of LC3-II/I and expression of Beclin-1 were significantly decreased, while the expression of SQSTM1/P62 was increased in the LPS group. Moreover, overexpression of PKD2 increased the LC3-II/I ratio and Beclin-1 level and reduced the SQSTM1/P62 level in the PKD2 overexpression plus LPS group. Importantly, the effect of PKD2 overexpression on autophagy-related protein levels in the lung tissue with LPS-induced ALI was reversed by 3-MA. Additionally, immunofluorescence analysis showed decreased SQSTM1/P62 levels in the lung tissue from the PKD2 overexpression + LPS group compared with the LPS group. The effect of PKD2 overexpression on the LPS-induced increase in SQSTM1/P62 levels in the lung tissue was offset by 3-MA (Fig. 4K). The above results indicated that overexpression of PKD2 reduced LPS-induced lung injury by activating autophagy.

## Discussion

We have verified the important ability of PKD2 overexpression to lessen the inflammatory response in vitro and in vivo. Collectively, these data provided evidence that overexpression of PKD2 increased the LC3-II/I ratio and reduces the SQSTM1/P62 level in lung epithelial cells and lung tissues exposed to LPS. Meanwhile, the suppressive effect of PKD2 overexpression against the inflammatory response and its ability to alleviate lung injury were reversed by 3-MA. This study is the first to show that autophagy caused by overexpression of PKD2 can alleviate lung epithelial cell injury in LPS-induced ALI. Our results provide evidence that clarifies the underlying mechanism by which overexpression of PKD2 acts as a regulator of lung epithelial cell function.

Autophagy has different effect in different models of lung injury generated in response to various stimuli. For example, one component of complement, C5a, is necessary for the full development of intestinal ischaemia/reperfusion (IR)-induced lung injury. C5a receptor (C5aR)-mediated autophagy induces alveolar macrophage apoptosis, disrupting pulmonary homeostasis and contributing to the development of ALI [22]. In the epithelium, the activation of autophagy may be beneficial in LPS-induced ALI. Inactivation of mechanistic target of rapamycin (MTOR [a serine/threonine kinase]) in the epithelium prevents LPS-induced ALI, likely through the activation of autophagy and subsequent inhibition of NF-κB activation [13]. Similar to the results of this study, our results demonstrated that inhibition of autophagy could aggravate the destruction of lung epithelial cell function and inhibit the protective role of overexpression of PKD2 against LPS-induced ALI, illustrating that autophagy positively modulates lung epithelial cell function under LPS insult. The underlying mechanisms by which autophagy exerts both deleterious and protective effects in lung epithelial cell injury remain unclear, and further research on the dual functions of autophagy in ALI is needed.

PKD2 (or PC2, TRPP1, formerly TRPP2), a membrane protein with six transmembrane segments, consists of 968 amino acids. PKD2 is primarily localized in the ER/sarcoplasmic reticulum (SR) and acts as a Ca^2+^-permeable, nonselective ion channel [23]. PKD2 was demonstrated to protect cardiomyocytes from apoptotic cell death under glucose starvation by increasing autophagy. This may be because PKD2 interacts with ryanodine receptor 2 (RyR2) to control Ca2^+^ release from the SR and further regulate the activity of AMP-activated protein kinase (AMPK) and mTOR, influencing autophagy and apoptosis [24]. PKD2 is a novel regulator of autophagy that was shown to act through ion channel-dependent control of intracellular Ca^2+^ homeostasis in a stress-induced cardiomyocyte model [25]. Consistently, PKD2 was necessary for autophagy induced by hyperosmotic stress in colon, cervical, and HeLa cancer cell lines [26]. Although some of these studies demonstrated that the loss of PKD2 function inhibited autophagy by increasing MTORC1 activity, PKD2 overexpression did not influence MTORC1 despite the increase in autophagy [24, 26–28]. Thus, the ability of PKD2 to regulate autophagy may involve MTORC1-independent mechanisms. PKD2 is usually found at mitochondria-associated membranes (MAMs), the ER and the primary cilium [29, 30]. Studies have shown that PKD2 interacts with BECLIN-1 to modulate autophagy. However, there is no evidence of a relationship between PKD2 and autophagy in sepsis-induced ALI. Here, we show that PKD2 is sufficient and necessary for autophagy induction. In our study, overexpression of PKD2 was sufficient to induce autophagy and further alleviate LPS-induced ALI.

In conclusion, the current study suggests that overexpression of PKD2 and subsequent activation of autophagy in lung epithelial cells was essential for alleviating LPS-induced lung epithelial cell injury in vitro and ALI in vivo. Taken together, these findings provide new perspectives on the correlation between PKD2 and autophagy and may provide novel targets for preventing ALI induced by LPS.

## Electronic supplementary material

Below is the link to the electronic supplementary material.

Supplementary Material 1

## Data Availability

All the data supporting the findings of this study are available from the corresponding author upon reasonable request.
