# Supplementary material for: PKD2/polycystin-2 inhibits LPS-induced acute lung injury in vitro and in vivo by activating autophagy
Source: BMC Pulm Med. 2023 May 18;23:171. doi: 10.1186/s12890-023-02449-w (PMC10193782; doi:10.1186/s12890-023-02449-w)
Supplement: Supplementary file 1 — Supplementary Material 1 [file 12890_2023_2449_MOESM1_ESM.docx]

Additional file 1:

**
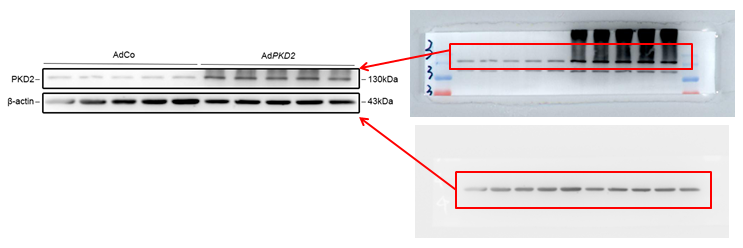
Figure S1. Full length blot for Figure 1A.** (A) Expression levels of PKD2 were determined by western blot in Adco-transfected or AdPKD2-transfected A549 cells. This image is also the Figure 1A in the manuscript. (B) The original images of full-length blots for A (Figure 1A).

A B

**Figure S2. Full length blot for Figure 2G**. A549 cells were transfected with AdCo or AdPKD2 for 48 h. Then, the cells were stimulated with LPS (50 μg/ml) for 12 h or pretreated with the autophagy inhibitor 3-MA (1 mol/L) for 15 min, followed by LPS (50 μg/ml) treatment for 12 h. (A) Expression levels of SQSTM1/P62 and Beclin-1 and the LC3-II/I were determined by western blot. This image is also the Figure 2G in the manuscript. (B) The original images of full-length blots for A (Figure 2G)


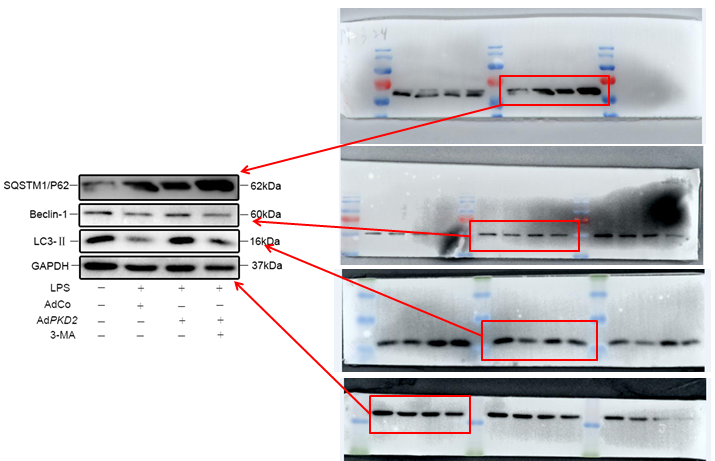
A B

**Figure S3. Full length blot for Figure 3B.** (A) Expression levels of PKD2 were determined by western blot in Adco-infected or AdPKD2-infected mice. This image is also the Figure 3B in the manuscript. (B) The original images of full-length blots for A (Figure 3B)


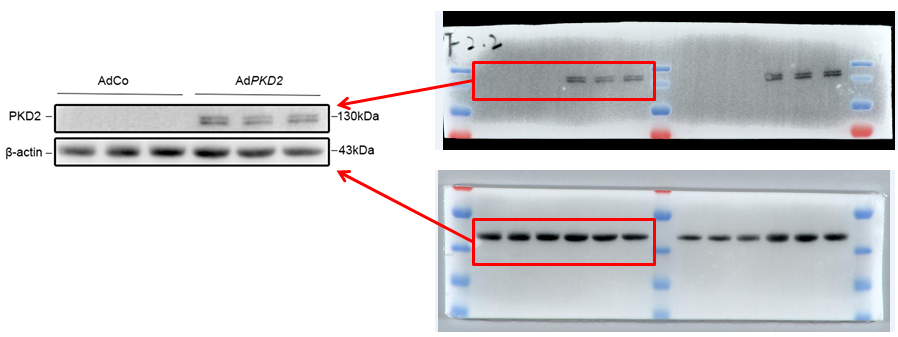
A B

**Figure S4. Full length blot for Figure 4G.** Mice were intratracheally injected with adenovirus expressing PKD2 or GFP (5×1012 vg/ml) in 30 μl of PBS for three days. Then, the mice were intratracheally injected with LPS (1 mg/kg) for 24 h or intraperitoneally injected with the autophagy inhibitor 3-MA (15 mg/kg) for 30 min, followed by LPS (1 mg/kg) treatment for 24 h. (A) Expression levels of SQSTM1/P62 and Beclin-1 and the LC3-II/I were determined by western blot. This image is also the Figure 4G in the manuscript. (B) The original images of full-length blots for A (Figure 4G)


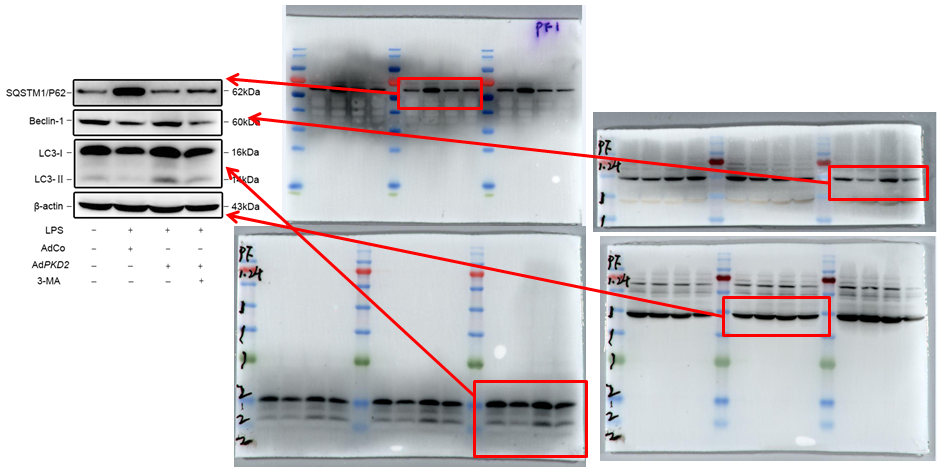
A B
